# Fulminant Type 1 Diabetes Mellitus With Concomitant Coxsackievirus B6 Antibody Elevation and RNA‐Based COVID‐19 Vaccination: A Case Report and Review of the Literature

**DOI:** 10.1002/ccr3.70429

**Published:** 2025-04-15

**Authors:** Hisaka Minami, Shinya Furukawa, Teruki Miyake, Keisho Kurokawa, Yasuhiro Sasaki, Masayuki Nakaishi, Kensuke Nagamatsu, Hiroka Yamago, Naozumi Shibata, Yoichi Hiasa, Bunzo Matsuura

**Affiliations:** ^1^ Department of Internal Medicine Ehime Prefectural Niihama Hospital Niihama Ehime Japan; ^2^ Health Services Center Ehime University Matsuyama Ehime Japan; ^3^ Department of Gastroenterology and Metabology Ehime University Graduate School of Medicine Toon Ehime Japan; ^4^ Department of Cardiology, Pulmonary, Hypertension and Nephropathy Ehime University Graduate School of Medicine Toon Ehime Japan; ^5^ Department of Cardiology Ehime Prefectural Niihama Hospital Niihama Ehime Japan; ^6^ Department of Urology Ehime Prefectural Niihama Hospital Niihama Ehime Japan; ^7^ Department of Internal Medicine Yawatahama City General Hospital Yawatahama Ehime Japan; ^8^ Department of Gastroenterology Ehime Prefectural Niihama Hospital Niihama Ehime Japan; ^9^ Department of Lifestyle‐Related Medicine and Endocrinology Ehime University Graduate School of Medicine Toon Ehime Japan

**Keywords:** autoimmune disease, COVID‐19, diabetes, type 1 diabetes, vaccine

## Abstract

Fulminant type 1 diabetes is a rare but severe condition that can develop rapidly, often in association with viral infections. In this case, coxsackievirus B6 antibody levels were elevated after disease onset, though its significance remains unclear. This highlights the importance of thorough viral screening in fulminant type 1 diabetes and the need for further research to better understand potential contributing factors, including in post‐vaccination contexts.

## 1 Introduction

Fulminant type 1 diabetes mellitus (FT1D) is characterized by almost normal hemoglobin A1c at onset, rapid onset with ketoacidosis, and the absence of islet‐associated autoantibodies [1, 2, 3]. The incidence of FT1D in the Asian population is higher compared to the Western population. In Japan, the percentage of FT1D was 19.4% among ketosis‐onset type 1 diabetes [4]. The etiology of FT1D is still not fully understood. However, a close association between viral infections and the onset of FT1D has been reported. Various viruses, including human herpesvirus 6, enterovirus, cytomegalovirus, rotavirus, and coxsackievirus, contribute to the onset of FT1D [1, 2, 3]. Coxsackievirus antibody elevation has been reported in patients with FT1D [5, 6, 7]. The underlying mechanisms linked to the coxsackievirus infection and the onset of type 1 diabetes mellitus have been proposed [8, 9, 10, 11, 12].

The COVID‐19 pandemic is a serious public health problem that has resulted in a significant number of deaths worldwide [13]. In response to the pandemic, more than 70% of the world population has been vaccinated against COVID‐19 [14]. During the rollout of COVID‐19 vaccination campaigns, reports emerged regarding the development of several autoimmune diseases including thyroid disease, autoimmune hepatitis, systemic lupus erythematosus, and FT1D after vaccination [15, 16, 17, 18, 19, 20, 21, 22, 23, 24, 25, 26]. However, the causal relationship between the vaccine and the onset of autoimmune diseases is not proven.

In the United States [27] and Hong Kong [28], the incidence of diabetes and type 1 diabetes is similar for those with and without COVID‐19 vaccination, respectively. Thus, we inferred that vaccination may not be associated with FT1D. However, to date, no studies have identified any viral infections associated with FT1D following COVID‐19 vaccination. Herein, we present a case of fulminant type 1 diabetes mellitus with concomitant coxsackievirus B6 antibody elevation and RNA‐based COVID‐19 vaccination. We also provide a review of the literature on cases of FT1D after the COVID‐19 vaccine.

## 2 Case History

A 58‐year‐old man received his second dose of the Moderna messenger ribonucleic acid (RNA) vaccine. Three days before his third vaccination, his blood glucose and HbA1c were 105 mg/dL and 5.9%, respectively. The day after his third vaccination, he had a high fever that lasted for only 1 day. Nine days after vaccination, he began to experience general fatigue, high fever, nausea, and thirst. He attended a clinic for appetite loss on the tenth day after vaccination. He was moved from the clinic to the emergency unit in Ehime Prefectural Niihama Hospital, presenting with severe appetite loss, abnormal electric cardiogram, and low systolic blood pressure.

## 3 Differential Diagnosis, Investigations, and Treatment

Upon admission, his temperature was 36.6°C and his consciousness level was drowsy (Glasgow Coma Scale: E4 V5 M6). His height, weight, and body mass index were 166.3 cm, 60 kg, and 21.6 kg/m^2^, respectively. His blood pressure, heart rate, and oxygen saturation (room air) were 60/40 mmHg, 75/min, and 90%, respectively. Acute renal failure with hyperkalemia was diagnosed, and treatment with calcium glucuronate administration and continuous hemodiafiltration was initiated. In addition, based on hyperglycemia and arterial blood gas analysis (Table 1), the patient was also diagnosed with diabetic ketoacidosis and received insulin and infusions. At the same time, he was diagnosed with FT1D based on his HbA1c, markedly decreased insulin secretion, other symptoms, and clinical course. The day after admission, the patient was able to eat, and he was discharged from the hospital on the tenth day.

**TABLE 1:** Laboratory data.

Peripheral blood		Reference range
White blood cell/mm^3^	11,200	3300–8600
Red blood cell, ×10^4^/mm^3^	396	435–555
Hemoglobin, g/dL	12.1	13.7–16.8
Hematocrit, %	42.2	40.7–50.1
Platelet, ×10^4^/mm^3^	23.4	15.8–34.8
Biochemistry		
Blood urea nitrogen, mg/dL	86	6–21
Creatinine, mg/dL	3.53	0.65–1.09
Sodium, mmol/L	120	136–148
Potassium, mmol/L	8.4	3.6–5
Chlorine, mmol/L	74	98–108
Plasma glucose, mg/dL	1499	80–110
Hemoglobin A1c, %	7.0	4.6–6.2
Fasting C‐peptide, ng/mL	0.1	0.8–2.5
Glucagon‐stimulate C‐peptide, ng/mL	0.1	
Urine C‐peptide (μg/day)	1.5	22.8–155.2
Immunological tests		
Anti‐GAD antibody, U/mL	< 5.0	< 5.0
Anti‐IA‐2 antibody, U/mL	< 0.6	< 0.6
Insulin autoantibody, U/mL	< 125.0	< 125.0
Arterial blood gas		
pH	7.002	7.350–7.450
pCO_2_, mmHg	20.2	32.0–48.0
pO_2_, mmHg	116	83.0–108.0
HCO_3_^−^, mmol/L	5.0	
Base excess, mmol/L	−24.7	
Lactate, mmol/L	7.2	
HLA‐DNA typing		
DRB1 04:05/08:03		
DQB1 04:01/06:01		

Abbreviations: GAD, glutamic acid decarboxylase;HLA, human leukocyte antigen; IA‐2, insulinoma‐associated protein‐2.

## 4 Outcome and Follow‐Up

His insulin regimen was basal bolus insulin therapy. To avoid hypoglycemia, continuous glucose monitoring (CGM) was started. He continued to undergo treatment with multiple daily insulin injection therapy with insulin lispro and insulin degludec as an outpatient. Several virus examinations were measured at the time of admission and the 21st day after admission. Coxsackie virus B6 antibody levels rose mildly on the 21st day after admission and rose slightly further 2 months later (Table 2). None of the other virus antibody titers showed any change in blood tests at admission or on day 21, so the third test in the second month was not performed.

**TABLE 2:** The results regarding virus infection.

	First test (at admission)	Second test (Day 21)	Third test (Two months)
Coxsackie A2 (NT)	32	16	
Coxsackie A3 (NT)	32	16	
Coxsackie A4 (NT)	64	32	
Coxsackie A5 (NT)	4	< 4	
Coxsackie A7 (NT)	< 4	< 4	
Coxsackie A9 (NT)	90	45	
Coxsackie A10 (NT)	64	64	
Coxsackie A16 (NT)	< 4	< 4	
Coxsackie B1 (NT)	256	256	
Coxsackie B2 (NT)	< 4	< 4	
Coxsackie B3 (NT)	< 4	< 4	
Coxsackie B4 (NT)	256	256	
Coxsackie B5 (NT)	8	8	
Coxsackie B6 (NT)	< 4	4	8
EB virus VCA‐IgM (IFA)	< 10	< 10	
EB virus VCA‐IgG (IFA)	160	160	
Parainfluenza B1 (HI)	< 10	< 10	
Parainfluenza B2 (HI)	< 10	< 10	
Parainfluenza B3 (HI)	10	10	
HHV‐6 (real time PCR)	Negative		
HHV‐7 (real time PCR)	Negative		
CMV‐IgM (CLIA)	< 0.85	< 0.85	
CMV‐IgG (CLIA)	< 6.0	< 6.0	

Abbreviations: CLIA, chemiluminescent immunoassay; CMV, cytomegalovirus; EB, Epstein–Barr; HHV, human herpes virus; HI, hemagglutination inhibition assay; IFA, indirect fluorescent antibody method; NT, neutralization test; PCR, polymerase chain reaction; VCA, virus capsid antigen.

## 5 Literature Review

A literature review was conducted using the PubMed database to search for case reports from January 2020 to December 2024 using the following search terms: (type 1 diabetes mellitus) AND (COVID‐19) AND (vaccination). Case reports not written in English were excluded. After an initial screening of titles and abstracts, full‐text articles were reviewed for relevance. This yielded 22 studies, of which nine studies were identified as relevant after carefully excluding case reports that did not meet the diagnostic criteria for fulminant type 1 diabetes.

## 6 Discussion

We reported elevation of antibody titer for coxsackievirus B6 in a case of FT1D after COVID‐19 vaccination.

We have summarized nine FT1D cases following COVID‐19 vaccination in Table 3. While two reports indicated that serological tests showed no evidence of viral infections [22, 26], other reports did not mention viral infections [18, 19, 20, 21, 23, 24, 25]. In general, the incidence of FT1D in Asian populations is higher compared to Western populations [1, 2, 3]. DRB1*0405 and DQB1*0401 and/or DRB1*0901 and DQB1*0303 were susceptible haplotypes for FT1D [1, 2, 3]. All Japanese patients, including the present case, had DRB1*0405 and DQB1*0401 alleles. Two (case 1 and case 7) of the nine patients had cancer and were on immune checkpoint inhibitors or steroids. In two cases with cancer, the development of FT1D might be an immune‐related adverse event. Race and HLA between post‐vaccination‐onset fulminant type 1 diabetes and previously reported fulminant type 1 diabetes are similar [1, 2, 3].

**TABLE 3:** Comparison of the case of fulminant type 1 diabetes mellitus after coronavirus disease‐2019 vaccination.

	Case 1	Case 2	Case 3	Case 4	Case 5	Case 6	Case 7	Case 8	Case 9	Case 10
Authors	Ohuchi, et al.	Sakurai, et al.	Lin, et al.	Kobayashi, et al.	Sasaki, et al.	Tang, et al.	Tanaka, et al.	Huang, et al.	Izumi, et al.	Present case
Published year	2021	2022	2022	2022	2022	2022	2023	2023	2023	2024
Population	Japan	Japan	Taiwan	Japan	Japan	China	Japan	Korean	Japan	Japan
Age, year	45	36	39	58	45	50	60	39	47	58
Sex	Male	Female	Female	Male	Female	Male	Male	Female	Male	Male
BMI	NA	NA	19.2	NA	20.6	18.1	NA	17.97	24.3	21.6
Vaccine	Pfizer	Pfizer	the recombinant spike protein vaccine Medigen/Pfizer	Pfizer	Pfizer	COVID‐19 inactived vaccine (CoronaVac)	Pfizer	Protein subunit vaccine (Zifivax)	Pfizer	Moderna
Number of vaccine administrations	Second	First	Second	Second	First	First	Third	Fourth	Third	Third
Time from vaccine to onset	3 days	3 days	14 weeks	15 weeks	3 days	6 days	2 days	4 days	1 day	9 days
Time from vaccine to hospital	3 days	7 days	14 weeks	15 weeks	6 days	7 days	2 days	6 days	4 days	11 days
Plasma glucose level, mg/dl	655	501	364	1514	344	Elevated	1000 or more	508	658	1499
HbA1c, %	NA	7.0	6.4	7.8	7.6	Elevated	NA	5.9	5.8	7.0
Plasma C‐peptide	0.09 ng/mL	0.35 ng/mL	< 0.02 ng/mL	< 0.03 ng/mL	0.33 ng/mL	0.1 ng/mL	NA	0.001 ng/mL	< 0.1 ng/mL	0.1 ng/mL
Serum pancreatic enzyme elevation	NA	(+)	NA	(+)	(+)	(+)	NA	(−)	(−)	NA
Diabetes family history	NA	(−)	(+)	(−)	NA	(+)	NA	(−)	(−)	(−)
		(−)		(−)						
Andi‐GAD body	(−)	(−)	Positive	Positive	(−)	(−)	NA	(−)	Positive	(−)
Anti‐IA‐2 body	(−)	(−)	(−)	(−)	(−)	(−)	NA	(−)	(−)	(−)
Anti‐ZnT8 body	NA	(−)	NA	(−)	(−)	(−)	NA	(−)	NA	NA
TPO body	NA	NA	NA	(−)	(−)	NA	NA	NA	(−)	(−)
Tg body	NA	NA	NA	(−)	(−)	NA	NA	NA	(−)	(−)
HLA DRB1	NA	04:05/08:03	NA	04:05/09:01	04:05:01/13:02:01	09:01/09:01	NA	NA	04:05:01/13:02:01	04:05/08:03
HLA DQB1	NA	04:01/06:01	NA	03:03/04:01	04:01:01/06:04:01	02:03/03:03	NA	NA	04:04:01/06:04:01	04:01/06:01
Virus infection	NA	NA	NA	NA	Parainfluenza (−)	NA	NA	NA		Parainfluenza (−)
					CMV (−)				CMV (−)	CMV (−)
					HHV (−)				HHV (−)	HHV (−)
					EBV (−)				EBV (−)	EBV (−)
					Coxsackie virus (−)				Coxsackie virus (−)	Coxsackie virus B6 (+)
Immune checkpoint inhibitor	(+)	(−)	(−)	(−)	(−)	(−)	(+)	(−)	(−)	(−)
Steroid	(−)	(−)	(−)	(−)	(−)	(−)	(+)	(−)	(−)	(−)

Abbreviations: BMI, body mass index; CMV, cytomegalovirus; EBV, Epstein–Barr virus; GAD, glutamic acid decarboxylase; HHV, human herpes virus; HLA, human leukocyte antigen; IA‐2, insulinoma‐associated protein‐2; ZnT8, zinc transporter 8.

The incidence of diabetes in COVID‐19 survivors who received COVID‐19 vaccinations experiences a reduced risk of new‐onset diabetes in US real‐world data [27]. Epidemiologic study shows no difference in the incidence of type 1 diabetes between vaccinated and unvaccinated individuals in Hong Kong [28]. It is already known that FT1D is more common in Asian races; similarly, FT1D after COVID‐19 vaccination was only found in Asian races. A Japanese Nationwide survey showed the percentages of FT1D were 19.4% among ketosis‐onset type 1 diabetes [4]. In a Korean study, the percentages of FT1D were 3.2% and 14.6% in newly diagnosed type 1 diabetes and ketosis‐onset diabetes, respectively [29]. However, no FT1D case was found in an Italian study of 82 patients with newly diagnosed type 1 diabetes [30], a U.S. study of 41 patients with ketosis‐prone diabetes [31], and an Indian study of 55 children with recent‐onset type 1 diabetes [32]. Thus, FT1D is common in the Asian population, especially in Japan. All 10 cases of FT1D following COVID‐19 vaccination were Asian, and seven of these were Japanese patients, although COVID‐19 vaccination has been used worldwide. The awareness of FT1D is very high in Japan, which might cause reporting bias. Thus, vaccination for COVID‐19 might not be an etiology for post‐vaccine‐onset FT1D.

The etiology of FT1D is not still fully understood. Several viral infections, including cytomegalovirus, mumps virus, and human herpes virus‐6, have been identified in patients with FT1D [1, 2, 3]. Previously, coxsackievirus antibody elevation was reported in patients with FT1D [5, 6, 7]. However, no previous viral infection is identified in post‐vaccinated FT1D; this is the first case in which coxsackievirus B6 antibody elevation was identified in a patient with FT1D, with or without vaccination against COVID‐19. Several studies provide a positive association between coxsackievirus B and the onset of type 1 diabetes mellitus [8]. Prospective cohorts provide the association between exposure to coxsackievirus B and type 1 diabetes mellitus progression [9, 10]. Coxsackievirus B itself directly infected beta cells [11]. Secondary antiviral immune responses to coxsackievirus B‐infected beta cells were reported [8, 11]. Furthermore, epitope mimicry between coxsackievirus B and beta cell epitopes was identified [12]. Before the onset of FT1D, the only symptom was fever. Most coxsackievirus infections are asymptomatic [33]. However, coxsackievirus B6 antibody titers were delayed and slightly elevated after the onset of FT1D. These delayed antibody elevations are not indicative of an acute viral infection at the onset of diabetes. Therefore, a causal relationship between coxsackievirus B and FT1D has not yet been established in this case.

To further investigate the etiology of FT1D, it is necessary to measure viral antibody titers in FT1D patients after the COVID‐19 vaccine and to accumulate further cases.

In conclusion, we showed the coxsackievirus B6 antibody elevation in FT1D cases following COVID‐19 vaccination. The accumulation of such cases will provide important information about the involvement of viral infection in the development of FT1D after vaccination.

## Author Contributions

**Hisaka Minami:** conceptualization, writing – original draft, writing – review and editing. **Shinya Furukawa:** formal analysis, writing – original draft, writing – review and editing. **Teruki Miyake:** writing – original draft. **Keisho Kurokawa:** data curation. **Yasuhiro Sasaki:** data curation. **Masayuki Nakaishi:** data curation. **Kensuke Nagamatsu:** data curation. **Hiroka Yamago:** data curation. **Naozumi Shibata:** data curation. **Yoichi Hiasa:** writing – review and editing. **Bunzo Matsuura:** writing – original draft.

## Consent

Written informed consent was obtained from the patient for publication of this case report, and the identity of the patient had been protected.

## Conflicts of Interest

S.F. has received honoraria for lectures and meetings from Mochida Pharmaceutical, Eli Lilly, and Mitsubishi Tanabe Pharma. Other authors declare no conflicts of interest.

## Data Availability

The raw data supporting the conclusions of this article will be made available by the authors, without undue reservation, to any qualified researcher.
